# Clinically Optimized Adult Height Prediction From Key Bone and Pubertal Stages: Prospective Validation to Adult Height

**DOI:** 10.1210/jendso/bvaf201

**Published:** 2025-12-10

**Authors:** Huahong Wu, Yaqin Zhang, Chengdong Yu, Yang Li, Wen Shu, Tao Li, Guimin Huang, Dongqing Hou, Fangfang Chen, Junting Liu, Shaoli Li, Xin’nan Zong

**Affiliations:** Department of Growth and Development, Capital Center for Children's Health, Capital Medical University, Capital Institute of Pediatrics, Beijing 100020, China; Department of Growth and Development, Capital Center for Children's Health, Capital Medical University, Capital Institute of Pediatrics, Beijing 100020, China; Department of Growth and Development, Capital Center for Children's Health, Capital Medical University, Capital Institute of Pediatrics, Beijing 100020, China; Department of Endocrinology, Genetics and Metabolism, Beijing Children's Hospital, Capital Medical University, National Center for Children's Health, Beijing 100020, China; Department of Growth and Development, Capital Center for Children's Health, Capital Medical University, Capital Institute of Pediatrics, Beijing 100020, China; Child Health Big Data Research Center, Capital Center for Children's Health, Capital Medical University, Capital Institute of Pediatrics, Beijing 100020, China; Child Health Big Data Research Center, Capital Center for Children's Health, Capital Medical University, Capital Institute of Pediatrics, Beijing 100020, China; Child Health Big Data Research Center, Capital Center for Children's Health, Capital Medical University, Capital Institute of Pediatrics, Beijing 100020, China; Department of Epidemiology, Capital Center for Children's Health, Capital Medical University, Capital Institute of Pediatrics, Beijing 100020, China; Child Health Big Data Research Center, Capital Center for Children's Health, Capital Medical University, Capital Institute of Pediatrics, Beijing 100020, China; Child Health Big Data Research Center, Capital Center for Children's Health, Capital Medical University, Capital Institute of Pediatrics, Beijing 100020, China; Department of Growth and Development, Capital Center for Children's Health, Capital Medical University, Capital Institute of Pediatrics, Beijing 100020, China

**Keywords:** key bone grade, pubertal stage, integrated model, predict adult height

## Abstract

**Context:**

Accurate adult height prediction remains a challenge in pediatric endocrinology. Traditional bone age (BA) based methods are time-consuming, software-dependent, and unreliable, while ignoring the critical effect of pubertal progression on growth potential.

**Objective:**

In this work we aimed to develop a clinically optimized model for adult height prediction by replacing traditional BA with key bone grades to quantify growth potential, integrating pubertal stages to account for pubertal-stage growth variations, and establishing a direct mapping between “key bone grades + pubertal stage” and height growth potential.

**Methods:**

A cross-sectional study was conducted in Beijing (2022-2023). We performed Tanner-Whitehouse 3/radius-ulna-short bone grading and pubertal staging including prepuberty, on puberty, and completing puberty. Spearman analysis identified key bone combinations most associated with BA and height. An integrated model combining bone grades and pubertal stage was developed and validated in an independent cohort followed to adult height.

**Results:**

Key Spearman correlation revealed strong correlations of the radius, ulna and metacarpal I grading with BA (ρ = 0.94-0.96), with bone combinations (ρ = 0.98-0.99) outperforming any single bones. Three types of bone combinations (radius + ulna, radius + metacarpal I, and radius + ulna + metacarpal I) integrating with pubertal stages demonstrated approximately equivalent predictive performance for adult height prediction. Considering bone representativeness and feasibility, we prefer to propose the radius + metacarpal I combination with puberty stages as the clinically optimized model for adult height prediction. Independent validation cohort confirmed superior accuracy of the proposed model vs traditional BA-based methods: Mean prediction error was reduced from 0.71 cm to 0.02 cm, while the proportion of predictions error of 3 cm or less increased from 66.9% to 73.5%.

**Conclusion:**

The integrated bone-puberty model significantly improves prediction accuracy by incorporating skeletal maturity and pubertal dynamics. Its streamlined 2-bone protocol offers a practical tool for growth monitoring and clinical decision-making.

Accurate adult height prediction is critical in pediatric endocrinology and growth management. Bone age (BA) assessment as the commonly used method for adult height prediction enables early identification of growth abnormalities [1, 2]. However, traditional BA assessment methods including the Greulich-Pyle (G-P) and Tanner-Whitehouse (TW) systems have some limitations. The G-P atlas method is the simplest to use; however, it is based on radiographic data from North American children collected decades ago and may introduce population bias when applied to contemporary Chinese children [3]. In contrast, the TW3 method, as the latest version of the TW series, is regarded as one of the gold standards for BA assessment due to its systematic and reproducible approach. It involves grading 20 bones (13 metacarpal-phalangeal bones plus 7 carpal bones) according to defined maturity stages, with the total score then converted into BA [4]. To better align with the growth pattern of Chinese children, the TW3-Chinese (TW3-C) method was developed, which further improves its applicability in this population [5]. However, this method involves manual grading of 20 bones, most of which are classified into 8 grades based on specific morphological features such as the shape, size, and degree of fusion of the ossification centers. With nearly 200 distinct diagnostic features across all bones and stages [4], the process is inherently complex and demands substantial expertise [6]. Moreover, contemporary BA-based prediction models, including artificial intelligence–assisted approaches, exhibit limitations in data reliability and generalizability [7, 8]. Therefore, developing simplified clinically practical methods for adult height prediction remains an imperative clinical need.

Most important, pubertal development is a key determinant of height prediction, but traditional prediction methods focus exclusively on BA while ignoring the dynamic effect of pubertal progression on growth potential. Children with identical BA may exhibit varying Tanner stages, with annual growth velocity differing by 4 to 6 cm between Tanner stages 1 and 3 [9, 10]. This oversight introduces apparent errors in growth potential estimation, potentially compromising clinical decisions (eg, growth hormone therapy initiation). In addition, existing models frequently lack robust external validation tracking to actual adult height.

To address these limitations, our previous research simplified Tanner-Whitehouse 3/radius-ulna-short bone (TW3-RUS) BA assessment by focusing on 3 key bones (radius, ulna, metacarpal I), reducing the evaluation from conventional 13 bones, and this simplified approach maintained 95.7% concordance with the standard assessment in a Chinese pediatric population [11]. Building on this foundation, we aimed to develop a clinically optimized model for adult height prediction by replacing traditional BA with key bone grades to quantify growth potential, integrating pubertal stages to account for pubertal-stage growth variations, and finally establishing a direct mapping between “key bone grades + pubertal stage” and height growth potential. Furthermore, we performed external validation of the model's predictive performance and clinical feasibility using an independent cohort that was tracked to final adult height.

## Materials and Methods

### Study Population

We conducted a cross-sectional population-based study in 10 educational institutions (kindergartens, primary/junior/senior high schools) in Beijing, China, between September 2022 and September 2023. To minimize confounding in BA and height prediction [12, 13], inclusion criteria were chronological age 6 years or older; clinically normal growth status (height SD score [HtSDS] between −2 and +2, and body mass index SD score [BMISDS] between −3 and +3); BA chronological age difference of 2 years or less; no history of endocrine, skeletal, chronic systemic diseases; and not having received any growth-related interventions. A total of 4374 children aged 6 to 18 years were enrolled. The protocol was approved by the ethics committee of the Capital Institute of Pediatrics (approval No. SHERLL2022043). Written informed consent was obtained from all parents/guardians and participants.

### Physical Examinations

Height was measured using a mechanical stadiometer (Harpenden Portable Stadiometer, Holtain Ltd) to the nearest 0.1 cm. Weight was measured via a 4-electrode bioelectrical impedance (H-Key350, Beijing Sihaihuachen Medical Equipment Co Ltd) to the nearest 0.1 kg. BMI was calculated as weight in kilograms divided by height in meters squared. HtSDS and BMISDS were calculated based on the Chinese national growth references for children and adolescents aged 0 to 18 years [14, 15].

### Pubertal Staging

Pubertal staging was assessed according to Tanner criteria [16]: Boys were staged G1 to G5 based on testicular volume and penile development, girls staged B1 to B5 by breast development. Participants were categorized into 3 pubertal stages: prepuberty (G1/B1), on-puberty (G2-G3/B2-B3), and completing puberty (G4-G5/B4-B5).

### Bone Age Assessment

Left-hand wrist radiographs were obtained using a low-dose mobile x-ray device (KBA-1, Mednova) [17]. Two senior pediatricians (W.H. and Z.Y.) independently graded 13 bones using the TW3-RUS method in a double-blind manner. The interrater consistency was 0.95, and no statistically significant difference was observed between the 2 reviewers. Therefore, final grades were the mean of the 2 grades and BA was then calculated using the mean grades.

### Key Bone Combination Grading

Building on our previous validation of a simplified BA assessment model in more than 5000 healthy children and adolescents [11], we established that 3 key bones (radius, ulna, and metacarpal I) provide a clinically practical alternatives to the traditional 13-bone evaluation. These 3 bones were categorized into an 8-stage scale in accordance with the grading rules of the TW3 method for assessing skeletal maturity.

Considering the widely recognized pivotal role of the radius as a key matching criterion in the G-P method [3] and its substantial weighting in the TW scoring system [4], we considered it essential for a simplified model. Particularly in late puberty, when the metacarpals, phalanges, and carpal bones have already matured, the epiphyseal fusion status of the radius becomes a decisive indicator for evaluating remaining growth potential. Thus, we defined the following bone combinations using these 3 key bones, and excluding combinations that omitted the radius, such as ulna + metacarpal I. All bone grades were calculated and rounded to the nearest 0.5 (eg, 3.3 and 3.7 were both recorded as 3.5):

radius + ulna grade = (radius grade + ulna grade)/2

radius + metacarpal I grade = (radius grade + metacarpal I grade)/2

radius + ulna + metacarpal I grade = (radius grade + ulna grade + metacarpal I grade)/3

### Adult Height Prediction

The traditional BA-based prediction uses the TW3-RUS standard adapted for Chinese children's growth patterns (TW3-C–RUS method) [5], using its derived percentage of final height achieved (%FHA): predicted adult height (PAH) = current height/(%FHA/100) [18].

In this study, using a cohort of 4374 children (2247 boys and 2127 girls), we replaced BA with key bone grades integrated with pubertal stages to directly map %FHA, which reflects consumed growth potential. We detailed the assessment of key bone grades in the “Key Bone Combination Grading” section and the pubertal staging method in the “Pubertal Staging” section. To integrate these 2 measures, we first grouped children by bone grade and then further subdivided them according to pubertal stage. For each resulting subgroup, the mean height and %FHA were computed and presented in cross-tabulations (Tables 1-3). The %FHA values in Table 3 represent a composite prediction indicator that incorporates both skeletal maturity and pubertal development. Ultimately, PAH = current height/(%FHA/100), with %FHA determined by the combined key bone grades and pubertal stages.

**Table 1.** Attained height (cm) stratified by key bone grades and pubertal stages in boys

	1.5	2.0	2.5	3.0	3.5	4.0	4.5	5.0	5.5	6.0	6.5	7.0	7.5	8.0
**Radius + Ulna**														
Total	119.6	121.1	127.1	131.7	134.5	137.1	141.3	144.4	149.3	158.1	164.8	168.8	174.1	
Prepuberty	119.6	121.1	127.1	131.7	134.5	137.1	141.3	144.0	148.1	154.0	157.3			
On puberty									155.1	159.0	164.3	167.2	171.8	
Completing puberty										165.6	167.8	171.2	174.6	
**Radius + Metacarpal I**														
Total				119.5	121.6	127.1	135.9	142.7	148.5	157.1	163.3	167.8	171.9	174.8
Prepuberty				119.5	121.6	127.1	135.9	142.7	147.6	152.1	156.9			
On puberty									153.7	159.4	163.4	166.8	170.4	172.7
Completing puberty										164.0	165.3	169.6	172.8	175.0
**Radius + Ulna + Metacarpal I**														
Total	114.0	119.5	124.9	130.5	133.8	138.1	142.0	145.0	150.3	159.5	164.8	169.1	174.1	
Prepuberty	114.0	119.5	124.9	130.5	133.8	138.1	142.0	144.5	148.9	153.0	158.4			
On puberty							147.9	160.9	155.1	160.2	164.4	167.6	172.1	
Completing puberty										164.0	167.6	171.3	174.5	

**Table 2.** Attained height (cm) stratified by key bone grades and pubertal stages in girls

	1.5	2.0	2.5	3.0	3.5	4.0	4.5	5.0	5.5	6.0	6.5	7.0	7.5	8.0
**Radius + Ulna**														
Total		115.9	121.3	125.0	127.2	129.4	132.6	136.4	139.7	147.6	155.1	159.2	161.2	
Prepuberty		115.9	121.0	124.4	126.5	128.7	130.9	133.2	135.0	142.7				
On puberty			130.7	133.7	133.8	132.8	135.9	139.9	141.1	146.4	151.8	155.9	161.9	
Completing puberty								146.8	147.4	150.7	156.2	159.5	161.2	
**Radius + Metacarpal I**														
Total					117.2	121.1	126.8	133.5	138.3	144.9	152.9	156.9	160.4	161.0
Prepuberty					117.2	121.1	126.3	131.4	135.2	141.3				
On puberty							132.2	137.3	139.9	144.3	151.0	155.2	160.9	158.2
Completing puberty									144.3	149.8	154.4	157.0	160.4	161.1
**Radius + Ulna + Metacarpal I**														
Total			119.2	123.2	126.2	129.4	132.1	136.3	140.1	147.7	154.9	159.2	160.9	
Prepuberty			119.2	122.7	125.5	128.9	130.9	133.5	135.9	146.2				
On puberty				130.7	133.5	132.3	135.2	139.6	141.3	147.0	151.8	161.6	158.5	
Completing puberty								146.2	147.9	149.5	156.1	159.0	161.0	

**Table 3.** Percentage of final height achieved corresponding to radius + metacarpal I grades and pubertal stages

	3	3.5	4	4.5	5	5.5	6	6.5	7	7.5	8
**Boys**											
Prepuberty	67.9	69.1	72.2	77.2	81.0	83.8	86.4	89.1			
On puberty						87.3	90.5	92.8	94.7	96.8	98.1
Completing puberty							93.1	93.9	96.3	98.1	99.4
**Girls**											
Prepuberty		72.6	75.0	78.2	81.4	83.7	87.5				
On puberty				81.9	85.0	86.6	89.3	93.5	96.1	96.9	98.0
Completing puberty						89.3	92.8	95.6	97.2	99.3	99.8

### Validation Cohort

An independent cohort of children who underwent BA and development assessments between 2010 and 2019 was included. These children met the inclusion criteria in the study population, received no growth interventions, and were followed to adult height. Finally, a total of 121 children (57 boys and 64 girls) aged 6 to 16 years were enrolled.

### Statistical Analysis

Data were analyzed using IBM SPSS Statistics version 22.0 (IBM) and R 4.4.1 (Posit Software). Spearman rank correlation was used to assess correlations between bone grades (single and combined), BA, and height, with greater correlation coefficient (ρ) indicating stronger correlations. Continuous data are presented as median and interquartile range. Mean heights by bone combination grade are listed in the tables; the corresponding %FHA was calculated as (mean height for bone maturity grade and pubertal stage group)/(mean adult height of the study population) × 100%. Paired *t* tests were used for between-group comparisons of continuous variables. Categorical data were presented as counts and proportions (n [%]), with between-group comparisons performed using chi-square tests. Two-sided *P* less than .05 was statistically significant.

In the validation cohort, agreements between PAH using different methods and actual adult height were evaluated using Bland-Altman analysis with calculation of mean difference and 95% limits of agreement. Given the known differences in growth patterns by sex, all results were presented separately for boys and girls.

## Results

### Characteristics of the Study Population

This study comprised 4374 children (2247 boys [51.4%] and 2127 girls [48.6%]), with a mean chronological age of 11.0 ± 3.0 years and BA of 10.9 ± 2.9 years. Pubertal stage distribution was as follows: 2092 prepuberty children (1408 boys and 682 girls), 947 on-puberty children (469 boys and 478 girls), and 1335 completing-puberty children (370 boys and 965 girls). Table 4 presents detailed anthropometric characteristics of height, weight, BMI, and BA stratified by age and sex.

**Table 4.** Distribution of bone age, height, weight, and body mass index in the study population by age and sex

Age, y	No.	BA, y	Height, cm	Weight, kg	BMI
**Boys**					
6	78	6.0 (5.4-6.6)	118.5 (115.9-121.7)	22.5 (20.0-24.9)	15.8 (14.6-16.9)
7	223	7.3 (6.6-7.9)	124.7 (121.6-128.4)	24.6 (22.2-28.5)	15.9 (14.8-17.7)
8	257	8.0 (7.4-8.7)	130.6 (126.9-134.3)	28.3 (24.0-34.0)	16.4 (15.1-19.3)
9	281	9.1 (8.5-9.9)	136.5 (132.3-140.1)	31.9 (28.1-39.4)	17.5 (15.5-20.4)
10	248	10.4 (9.4-11.0)	142.5 (138.8-145.5)	37.5 (31.6-44.4)	18.4 (15.8-21.5)
11	216	11.2 (10.7-11.6)	147.7 (143.1-152.5)	42.8 (36.0-52.2)	20.0 (16.9-23.3)
12	221	12.0 (11.4-13.2)	155.7 (150.1-160.5)	48.6 (42.0-58.0)	20.8 (17.5-23.6)
13	219	13.3 (12.7-13.7)	164.5 (158.6-167.8)	55.9 (46.6-65.0)	20.5 (17.4-24.0)
14	172	14.1 (13.3-14.9)	168.9 (164.4-173.4)	61.2 (52.2-71.0)	21.4 (18.6-24.7)
15	140	15.1 (14.5-15.5)	172.8 (168.5-177.1)	63.8 (56.0-80.5)	21.4 (18.6-26.7)
16	67	15.5 (15.3-16.0)	174.4 (169.8-177.9)	70.4 (60.7-82.3)	23.0 (20.5-27.3)
17-18	125	16.0 (16.0-16.0)	175.5 (172.1-180.6)	68.5 (59.4-82.1)	22.4 (19.7-26.0)
**Girls**					
6	71	5.6 (5.2-6.5)	118.3 (114.3-121.3)	21.2 (19.0-23.6)	15.1 (14.1-16.2)
7	222	6.8 (5.9-7.6)	123.2 (120.3-126.4)	23.4 (21.3-25.7)	15.4 (14.3-16.7)
8	249	8.1 (7.4-8.7)	129.7 (126.6-133.3)	26.5 (23.7-30.0)	15.7 (14.5-17.4)
9	235	8.9 (8.2-9.5)	135.1 (130.5-139.2)	29.2 (25.5-34.2)	16.0 (14.6-17.8)
10	215	9.9 (9.3-10.8)	141.6 (136.8-145.9)	34.9 (29.7-40.8)	17.1 (15.4-19.8)
11	202	11.1 (10.3-11.5)	149.5 (144.9-154.4)	39.8 (34.4-47.5)	17.6 (16.1-20.8)
12	209	11.9 (11.5-12.6)	156.3 (152.5-160.0)	46.8 (41.9-52.3)	19.4 (17.1-21.6)
13	229	12.8 (12.3-13.2)	158.9 (155.5-162.6)	51.4 (45.5-58.5)	20.4 (18.1-22.8)
14	192	13.5 (12.9-13.8)	160.5 (157.0-164.3)	51.7 (45.6-59.1)	19.9 (17.8-22.9)
15	161	13.8 (13.6-15.0)	162.0 (158.1-164.9)	53.9 (46.8-61.6)	20.2 (18.1-23.1)
16	99	15.0 (13.8-15.0)	162.6 (159.3-165.7)	55.3 (48.2-62.8)	21.4 (18.2-23.6)
17-18	43	15.0 (15.0-15.0)	162.1 (158.5-165.6)	56.9 (49.6-64.0)	21.5 (19.0-24.8)

Data are presented as median (interquartile range).

Abbreviations: BA, bone age; BMI, body mass index.

### Correlations of Key Bones and Their Combinations With Bone Age and Height

**Figure 1.**
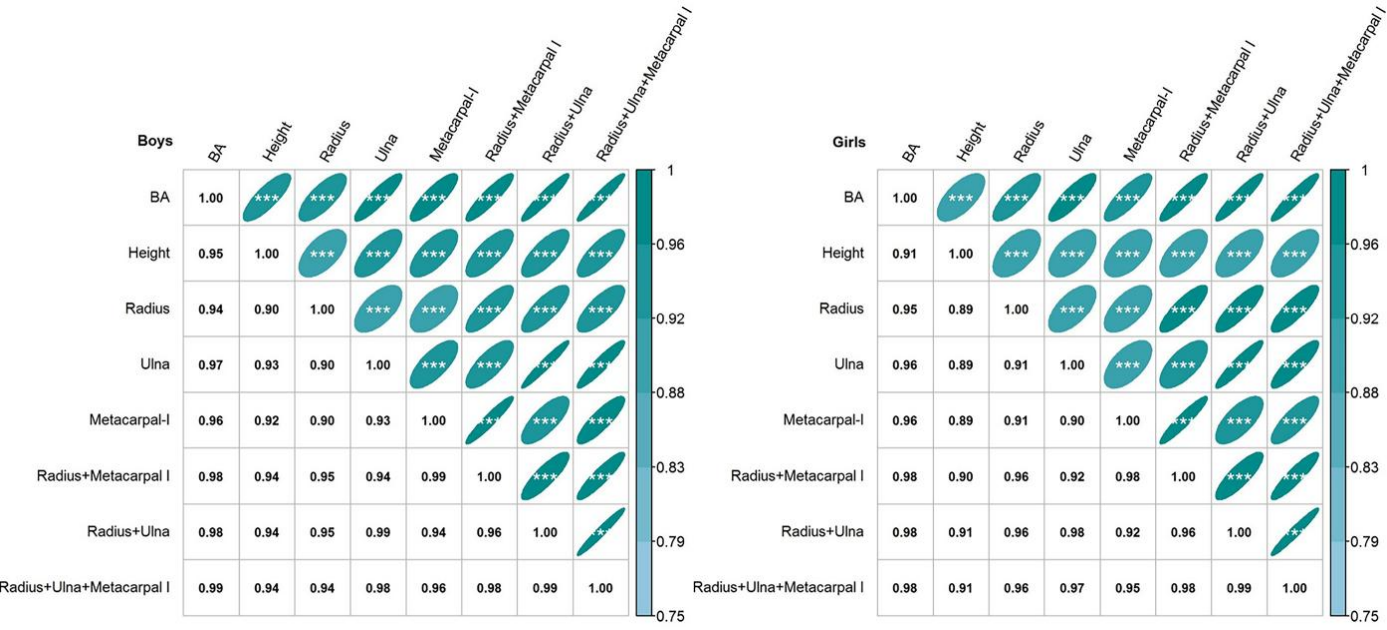
Spearman correlation coefficients of individual and combined key bone grades with bone age (BA) and height. *P less than .05; **P less than .01; ***P less than .001.

Spearman correlation analysis demonstrated strong correlations of key bone grades (radius, ulna, metacarpal I) and their combinations (radius + ulna, radius + metacarpal I, radius + ulna + metacarpal I) both with BA and height. Single bones showed high correlations with BA (ρ = 0.94-0.96) and height (ρ = 0.89-0.94), while bone combinations exhibited significantly stronger correlations (BA: ρ = 0.98-0.99; height: ρ = 0.90-0.94) (Fig. 1), with almost no differences observed among these 3 combinations.

These findings support that bone combinations provide superior predictive accuracy compared to any single bones. Therefore, in this study, we excluded single bones models and focused on bone combinations in subsequent analyses. By comparing achieved height and %FHA stratified by bone grades and pubertal stages, we evaluated the prediction outcomes of different bone combinations to identify the optimal clinical model for adult height prediction.

### Height Variation by Key Bone Grades + Pubertal Stages

Tables 1 and 2 present the mean achieved heights stratified by key bone grades + pubertal stages. Height consistently increased with advancing bone grade (increments of 1.5 to 16.0 cm per grade). Boys and girls both exhibited accelerated growth from grades 4 to 5 (height gains ≥10 cm), followed by marked deceleration after grade 7 and stabilizing near adult height at grade 8. Notably, within the same bone grades, pubertal stage advancement was associated with clinically significant height variations of 0.5 to 9.7 cm, demonstrating the independent contribution of pubertal development to growth potential beyond bone maturity.

### Growth Potential Variation by Key Bone Grades + Pubertal Stages

**Figure 2.**
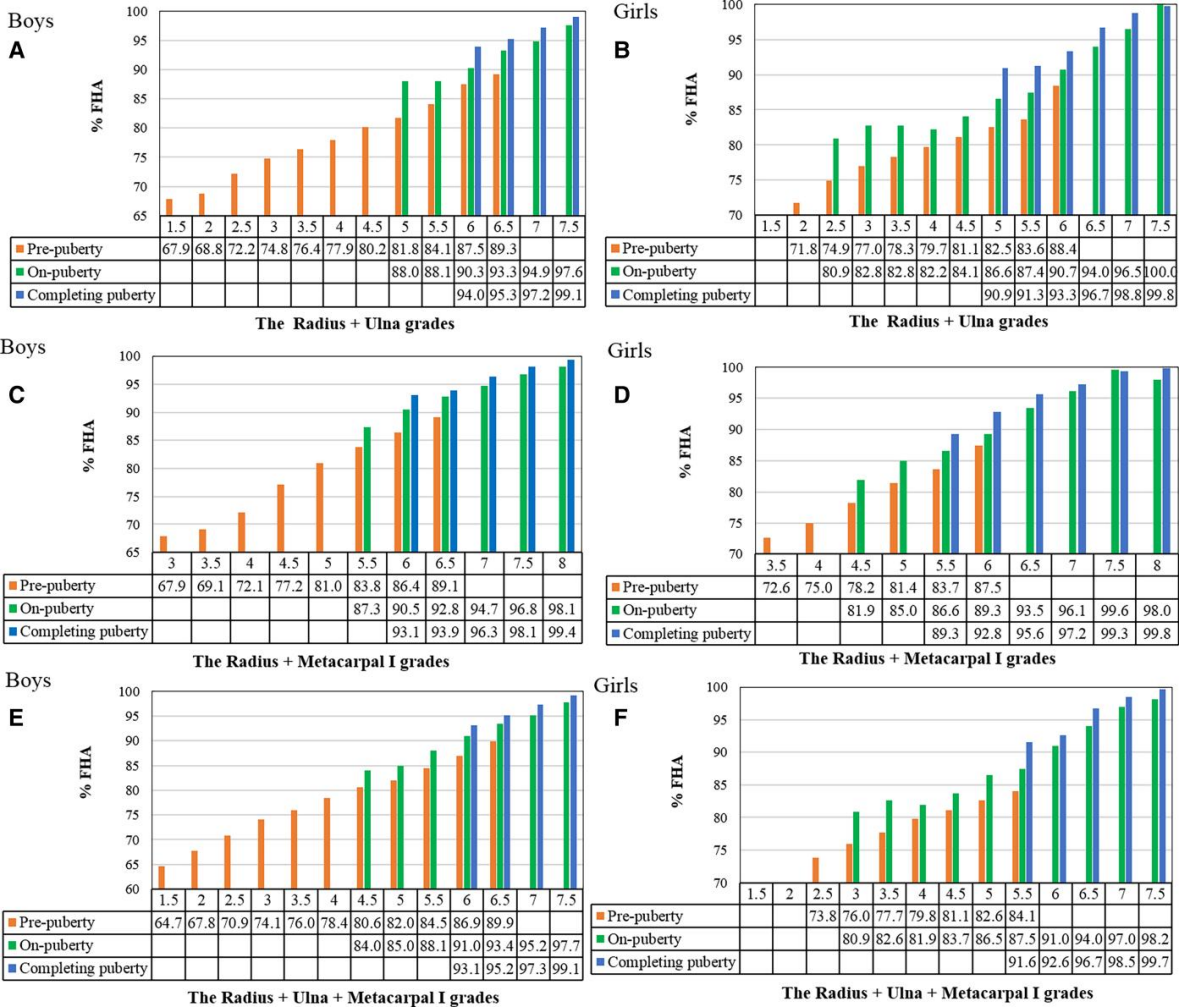
%FHA by combined bone grades and pubertal stages in boys and girls. A and B are %FHA for radius + ulna grades in boys and girls; C and D are %FHA for radius + metacarpal I grades in boys and girls; E and F are %FHA for radius + ulna + metacarpal I grades in boys and girls. %FHA, percentage of final height achieved.

The %FHA (reflecting consumed growth potential) was calculated as the percentage of achieved height (see Tables 1 and 2) to the mean adult height of the study population (175.5 cm for males, 162.1 cm for females). Fig. 2 illustrates that the %FHA progressed consistently across bone combination grades and pubertal stages. All bone combinations exhibited accelerated growth and rapid depletion of growth potential between grades 4 and 5, with %FHA increasing by up to 8.9% per grade. Growth deceleration occurred after grade 7, with %FHA increments consistently less than 4% per grade. Notably, within the same bone grades, %FHA increased by 3% to 7% from prepuberty to on-puberty and by 5% or less from on-puberty to completing puberty, confirming that pubertal progression independently modulates growth potential beyond the influence of bone maturity.

### Prediction Accuracy by Integrated “Key Bone Grades + Pubertal Stage” Model

**Figure 3.**
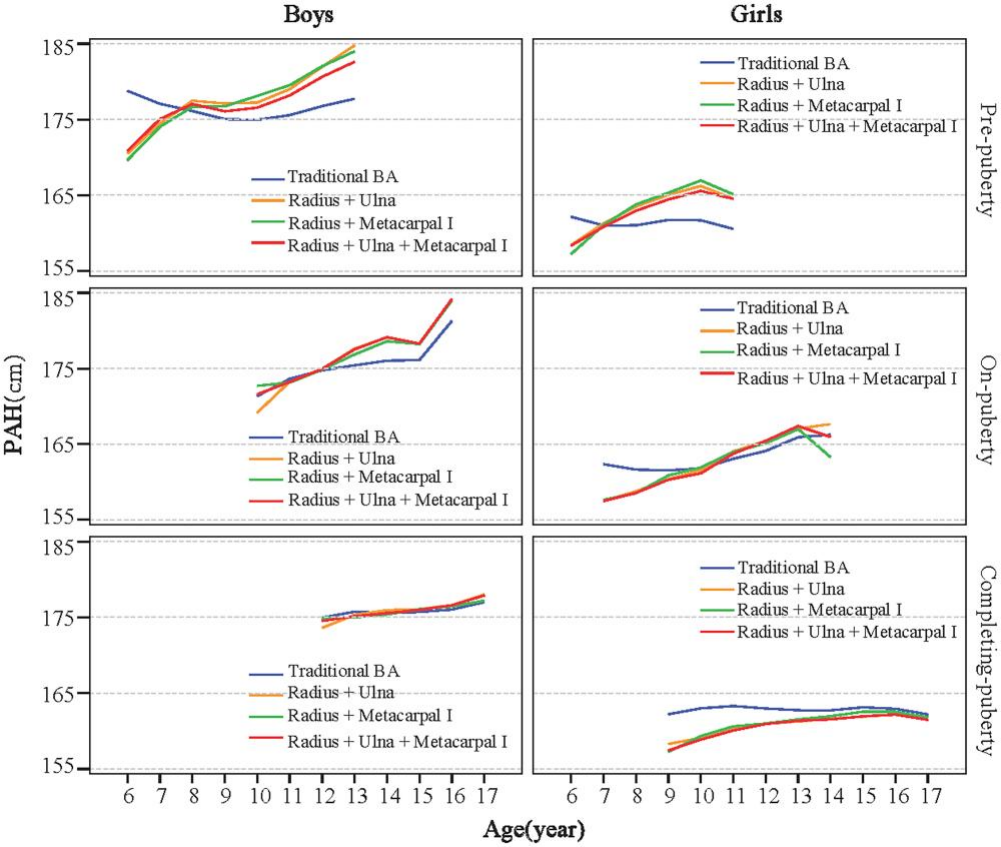
Prediction accuracy of 4 height-prediction models by pubertal stages in boys and girls. All models in this figure integrate key bone grades with pubertal stages. PAH, predicted adult height.

PAH was calculated for all participants using both the integrated model (%FHA values mapped to bone combination grade and pubertal stage in Fig. 2) and the traditional TW3-BA–based method. As shown in Fig. 3, the integrated model yielded higher PAH than traditional methods for later-puberty children (12-year-old boy or 10-year-old girl still in the prepuberty stage) but lower PAH for early-puberty girls (10-year-old already in the completing-puberty stage). Among these 3 bone combination + pubertal stages models, no notable differences were observed in their predicted outcomes.

### Our Proposed Clinically Optimized Predicted Adult Height Model

Given the equivalent predictive accuracy between 2- and 3-bone combinations integrated with pubertal stages models (see Fig. 3), we prefer to propose the radius + metacarpal I combination with pubertal stages model as the optimized clinical protocol. This protocol achieves an optimal balance between diagnostic efficiency and anatomical representativeness—incorporating both long bone (radius) and hand skeletal maturity (metacarpal I)—while maintaining practicality for routine clinical application.

#### Application protocol

The application protocol is as follows: (1) calculate the mean grades of the radius and metacarpal I; (2) locate the corresponding %FHA in the Table 3 based on bone grades and pubertal stages; and (3) compute PAH = current height/(%FHA/100).

### Prospective External Validation of the Proposed Model in Real-World Data

**Figure 4.**
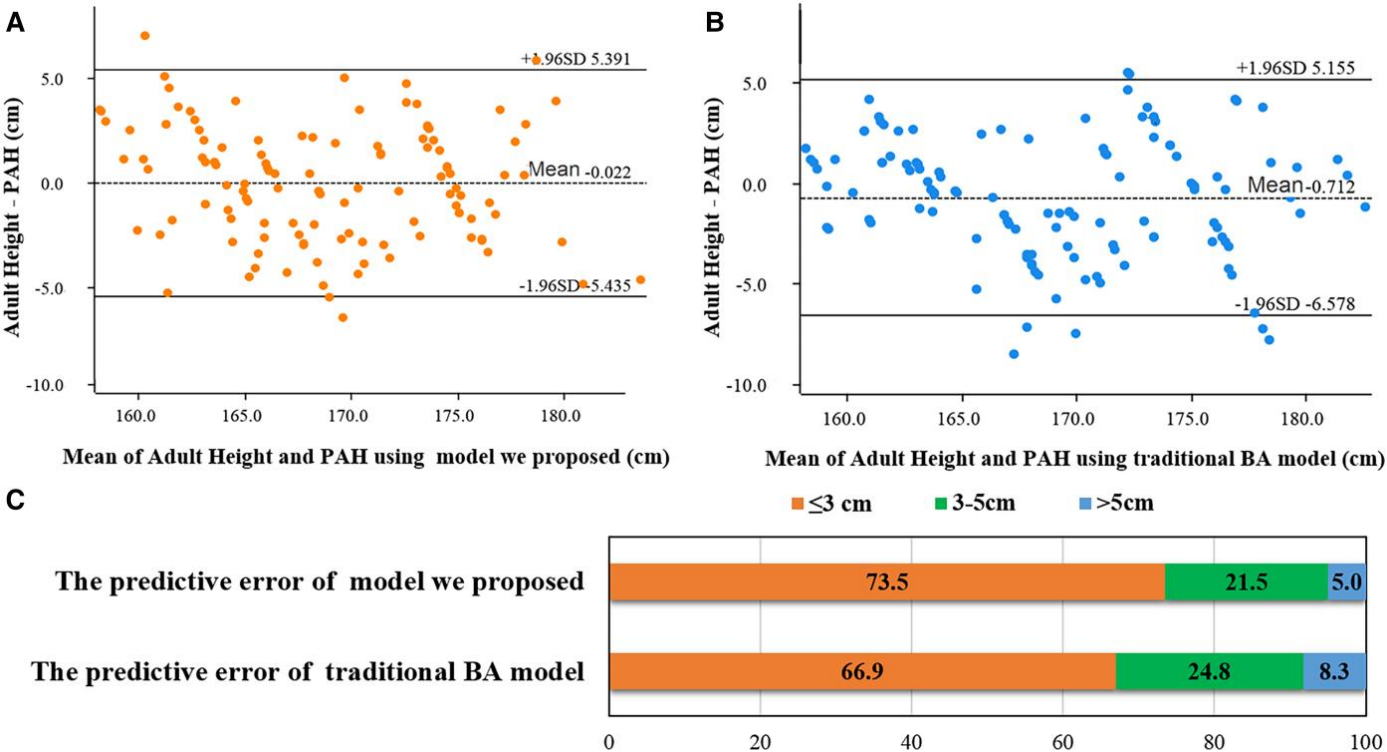
Agreement and error between PAH and actual adult height in the invalidation cohort. A is Bland-Altman analysis for actual adult height and PAH using the model we proposed; B is Bland-Altman analysis for actual adult height and PAH using traditional BA method. C is the predictive error of these 2 models. BA, bone age; PAH, predicted adult height.

The validation cohort included 121 participants (baseline characteristics presented in Table 5). Pairwise comparisons revealed no statistically significant difference between PAH and actual adult heights for the proposed model (*P* > .05), while the traditional TW3-C–RUS method showed significant discrepancy (*P* = .010). Bland-Altman analysis further confirmed these findings: For the proposed model, the mean difference from actual adult height was −0.02 cm (SD 2.76 cm), and 95% limits of agreement ranged from −5.4 to 5.4 cm, with a 73.5% of error less than or equal to 3 cm. For the traditional TW3-C–RUS method, the mean difference was −0.71 cm (SD 2.99 cm), with 95% limits of agreement from −6.6 to 5.2 cm, with a 66.9% of error less than or equal to 3 cm (Fig. 4).

**Table 5.** Characteristics of the validation cohort

	Boys (n = 57)	Girls (n = 64)
**Baseline characteristics in childhood**		
Age, y	12.0 (11.2 to 12.9)	10.0 (8.8 to 11.4)
Height, cm	159.2 (152.0 to 164.4)	144.4 (135.0 to 153.4)
Weight, kg	46.0 (40.8 to 51.0)	35.2 (29.0 to 42.5)
BMI	18.0 (16.9 to 20.5)	16.9 (15.8 to 18.8)
HtSDS	0.40 (0.05 to 0.90)	0.60 (0.10 to 1.38)
BMISDS	0.20 (−0.34 to 1.60)	0.72 (−0.18 to 1.70)
BA, y	13.6 (13.2 to 14.0)	11.2 (9.6 to 11.9)
Pubertal stage		
Prepuberty	14 (24.6)	24 (37.5)
On- puberty	37 (64.9)	33 (51.6)
Completing puberty	6 (10.5)	7 (10.9)
Mid-parent height	168.2 (167.5 to 170.5)	160.1 (158.9 to 162.0)
PAH using traditional BA	174.7 (171.7 to 177.8)	164.0 (160.9 to 168.5)
PAH using model we proposed	173.9 (171.0 to 176.9)	164.4 (160.6 to 167.0)
**Outcome assessment in adulthood**		
Age, y	23.2 (21.0 to 25.0)	20.6 (18.8 to 21.5)
Actual adult height, cm	175.0 (172.0 to 175.0)	163.5 (162.5 to 166.0)

Continuous variables are presented as median (interquartile range), and categorical variables as n (%): Prepuberty indicates Tanner stage G1 or B1; on-puberty indicates Tanner stage G2 to G3 or B2 to B3; completing puberty indicates Tanner stage G4 to G5 or B4 to B5.

Abbreviations: BA, bone age; BMISDS, body mass index SD score; HtSDS, height SD score; PAH, predicted adult height.

**Figure 5.**
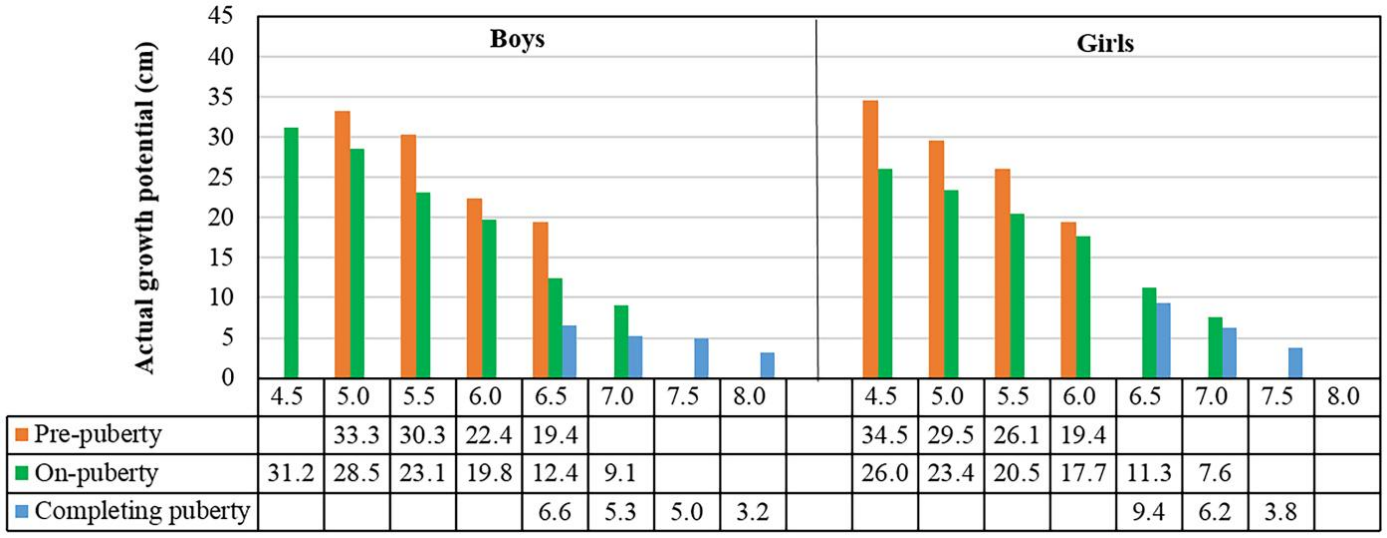
Actual residual height growth potential (cm) by radius + metacarpal I grades and pubertal stages in validation cohort.

To further validate the effect of bone maturity and pubertal development on residual growth potential using real-world data, we quantified the actual growth potential by radius + metacarpal I and pubertal stage in the independent validation cohort. Fig. 5 shows each increment in bone grade consumed 2.2 to 7.4 cm of residual growth potential, while pubertal stage advancement consumed 1.4 to 8.5 cm within the same bone grades. Notably, late-pubertal children exhibited statistically significantly greater residual growth potential than their early-pubertal counterparts across all bone grades (*P* < .001).

## Discussion

In this study, we established a quantitative framework integrating key bone grades (radius + metacarpal I) with pubertal stages for adult height prediction. This model directly addresses recognized limitations of traditional BA-based methods, which often overlook dynamic pubertal influences on growth potential. Our findings reveal that this integrated model not only achieves significantly improved predictive accuracy but also enhances clinical utility. This protocol (see Table 3) provides clinicians a practical tool for growth monitoring and evidence-based decision-making in pediatric practice.

### Key Bone Combination as an Efficient Alternative for Streamlined Adult Height Prediction

The traditional TW3-RUS BA assessment requires grading 13 bones. In our prior study, we identified key bones to simplify this process while maintaining diagnostic accuracy [11]. Building on this foundation, we further demonstrate a 2-bone combination (radius + metacarpal I) exhibits strong correlations both with BA and height (see Tables 1 and 2), which enables its direct substitution for comprehensive BA assessment in growth potential mapping. Anatomically, the radius captures longitudinal growth potential, while metacarpal I reflects hand bone maturity; this combination provides a multidimensional maturity assessment comparable to traditional BA evaluation. Clinical validation confirms physiologic relevance: Bone grades 4 to 5 in girls corresponded to approximately 9 years of age, coinciding with pubertal onset and accelerated height velocity in Chinese girls [19, 20], and marked growth deceleration observed after grade 7.

Ultimately, this approach streamlines the prediction workflow by directly mapping “key bone grades + pubertal stage” to %FHA, avoiding cumulative errors inherent in multistep conversions [21]. This novel, integrated model outperformed traditional BA-based prediction in accuracy (73.5% vs 66.5% of predictions errors ≤3 cm) and demonstrated comparable or superior performance to referenced machine-learning approaches [22, 23].

### Quantifying Pubertal Effect on Growth Potential and Enhancing Prediction Accuracy

Our analysis demonstrated that pubertal progression significantly modulates growth potential independent of bone maturity. Among boys with radius + metacarpal I grade 6, we observed an 11.9-cm height difference and 6.7% disparity in residual growth potential between prepubertal and completing pubertal stages (see Table 1 and Fig. 2). Each advancement in pubertal stage consumed 3% to 7% of residual growth potential, directly quantifying the effects of sex hormone on height growth [24, 25]. And, integrating bone grades with pubertal stages further enabled refined stratification of growth potential. As shown in Fig. 5, boys with radius + metacarpal I grade 5 had 4.8-cm greater residual growth potential at the on-pubertal stage vs the completing-pubertal stage (33.3 cm vs 28.5 cm). Consequently, our dual-parameter model achieved superior agreement with actual adult heights compared with traditional methods, particularly in early or late puberty (see Fig. 4) [16]. These findings confirm that integrating bone maturity with pubertal stages enhances height prediction accuracy beyond traditional BA methods.

### Clinical Feasibility and Application Demonstration

Our proposed model requires only height, grading of radius + metacarpal I, and Tanner stage, with PAH easily obtained from lookup tables (see Table 4), for instance, a 10-year-old girl (140.5 cm) with radius grade 6, metacarpal I grade 5 (combined grade 5.5), and on-pubertal stage (stage B2). Table 3 shows %FHA = 86.6%, yielding PAH = 140.5 cm/(86.6/100) = 162.2 cm. This efficient approach requires less than 3 minutes, making it feasible both for primary care and large-scale screening.

### Strengths and Limitations

Our study has several strengths. First, we established a novel, optimized, stratified model that quantifies growth potential by simplified combinations of key bones' grades + pubertal stages, both of which are objectively measured parameters. This integrated model streamlines the clinical assessment process and highlights puberty's dynamic influence on growth potential. Second, external validation in an independent cohort confirmed superior agreement between PAH derived from our proposed model and actual adult height compared to traditional BA-based methods. However, our study also has several limitations. First, as our established model was developed based on a Chinese pediatric population, its generalizability across counties required further evaluation. Furthermore, the current findings are potentially applicable only in settings that use the TW3 method and can ensure reliable assessment of pubertal status, such as pediatric endocrine clinics. Second, considering the validation cohort consisted of relatively healthy children in this study, prediction accuracy in special populations (eg, precocious puberty, obesity) requires further calibration [26, 27].

In conclusion, the “key bone grades + pubertal stages” height prediction model achieves an optimal balance of predictive accuracy and clinical practicality, offering a clinician-friendly tool for childhood growth monitoring and clinical decision-making. Further validations across diverse pediatric populations are warranted to confirm its generalizability and optimize region-specific applicability.

## Data Availability

The data are not publicly available due to privacy and informed consent concerns. Deidentified data supporting specific results may be available on reasonable request to the corresponding author.

## Abbreviations

BA: bone age
BMI: body mass index
BMISDS: body mass index SD score
%FHA: percentage of final height achieved
G-P: Greulich-Pyle
HtSDS: height SD score
PAH: predicted adult height
TW3-C: Tanner-Whitehouse 3–Chinese
TW3-RUS: Tanner-Whitehouse 3/radius-ulna-short bone
